# Prediction of radiation-induced acute skin toxicity in breast cancer patients using data encapsulation screening and dose-gradient-based multi-region radiomics technique: A multicenter study

**DOI:** 10.3389/fonc.2022.1017435

**Published:** 2022-11-10

**Authors:** Huichun Feng, Hui Wang, Lixia Xu, Yao Ren, Qianxi Ni, Zhen Yang, Shenglin Ma, Qinghua Deng, Xueqin Chen, Bing Xia, Yu Kuang, Xiadong Li

**Affiliations:** ^1^Medical Imaging and Translational Medicine Laboratory, Hangzhou Cancer Center, Hangzhou, China; ^2^Patient follow-up center, Hangzhou Cancer Hospital, Hangzhou, China; ^3^Department of Radiotherapy, Affiliated Hangzhou Cancer Hospital, Zhejiang University School of Medicine, Hangzhou, China; ^4^Department of Radiology, Hunan Cancer Hospital, Affiliated Cancer Hospital of Xiangya School of Medicine, Central South University, Changsha, China; ^5^Department of Radiotherapy, Xiangya Hospital Central South University, Changsha, China; ^6^Medical Oncology, Xiaoshan Hospital Affiliated to Hangzhou Normal University, Hangzhou, China; ^7^Medical Physics Program, University of Nevada, Las Vegas, NV, United States

**Keywords:** Breast cancer, radiation therapy, radiation-induced skin toxicity, machine learning, radiomics, gradient boosting decision tree

## Abstract

**Purpose:**

Radiation-induced dermatitis is one of the most common side effects for breast cancer patients treated with radiation therapy (RT). Acute complications can have a considerable impact on tumor control and quality of life for breast cancer patients. In this study, we aimed to develop a novel quantitative high-accuracy machine learning tool for prediction of radiation-induced dermatitis (grade ≥ 2) (RD 2+) before RT by using data encapsulation screening and multi-region dose-gradient-based radiomics techniques, based on the pre-treatment planning computed tomography (CT) images, clinical and dosimetric information of breast cancer patients.

**Methods and Materials:**

214 patients with breast cancer who underwent RT between 2018 and 2021 were retrospectively collected from 3 cancer centers in China. The CT images, as well as the clinical and dosimetric information of patients were retrieved from the medical records. 3 PTV dose related ROIs, including irradiation volume covered by 100%, 105%, and 108% of prescribed dose, combined with 3 skin dose-related ROIs, including irradiation volume covered by 20-Gy, 30-Gy, 40-Gy isodose lines within skin, were contoured for radiomics feature extraction. A total of 4280 radiomics features were extracted from all 6 ROIs. Meanwhile, 29 clinical and dosimetric characteristics were included in the data analysis. A data encapsulation screening algorithm was applied for data cleaning. Multiple-variable logistic regression and 5-fold-cross-validation gradient boosting decision tree (GBDT) were employed for modeling training and validation, which was evaluated by using receiver operating characteristic analysis.

**Results:**

The best predictors for symptomatic RD 2+ were the combination of 20 radiomics features, 8 clinical and dosimetric variables, achieving an area under the curve (AUC) of 0.998 [95% CI: 0.996-1.0] and an AUC of 0.911 [95% CI: 0.838-0.983] in the training and validation dataset, respectively, in the 5-fold-cross-validation GBDT model. Meanwhile, the top 12 most important characteristics as well as their corresponding importance measures for RD 2+ prediction in the GBDT machine learning process were identified and calculated.

**Conclusions:**

A novel multi-region dose-gradient-based GBDT machine learning framework with a random forest based data encapsulation screening method integrated can achieve a high-accuracy prediction of acute RD 2+ in breast cancer patients.

## 1 Introduction

Surpassing lung cancer as the leading cause of global cancer incidence, breast cancer accounted for 11.7% of all new cancer cases with 685,000 deaths, ranking the fifth leading cause of cancer mortality worldwide in 2020 (1). Most patients with breast cancer are treated with surgery (e.g., lumpectomy or mastectomy) followed by radiation therapy (RT) on the residual ipsilateral breast or chest wall, with alternative dose boost to the tumor bed and/or regional lymph node irradiation applied (2–4). Treatment-induced acute skin toxicity (i.e., acute radiodermatitis) with a different degree, ranging from erythema to desquamation (dry or moist), ulceration, and necrosis, is one of the most common acute side effects of RT underwent by breast cancer patients, with approximately 90% of treated patients experiencing erythema and 30% experiencing moist desquamation (5–8). Such acute skin toxicity negatively affects multiple aspects of quality of life (QOL) of breast cancer radiotherapy patients, such as physical discomfort, emotional distress, and body image disturbance, and so on (9).

The acute skin reactions are prone to progress during the treatment and remain after completion of the treatment. In addition, severe acute reactions may be prodromal of subsequent late effects (10), and the RT schedule might be changed or even terminated due to these negative reactions. Therefore, early prediction of acute radiodermatitis when formulating a radiation therapy regimen could potentially reduce the risk of skin toxicity. Furthermore, early management of acute radiodermatitis in breast cancer patients can improve both day-to-day functioning and satisfaction with radiation treatment, and therefore QOL and outcome of patients.

Qualitative evaluation of acute skin toxicity mainly by visual inspection of the skin-related symptoms of breasts is subject to practitioner bias, variability in grading dermatitis as well as differentiating the severe dermatitis (e.g., moist desquamation) due to clinician expertise, and underreporting by patients (9, 11). Most importantly, this method detects early signs of dermatitis with low sensitivity and specificity. Based on the semi-quantitative analysis of clinical and dosimetric predictors of acute skin toxicity, the normal tissue complication probability (NTCP) models can be established to predict severe acute skin toxicity in breast cancer patients (10). However, the prediction performance was relatively poor with an area under the curve (AUC) as low as 0.77 (10).

To improve the prediction performance, quantitative early thermal imaging biomarkers were identified and used in machine learning frameworks (i.e., thermoradiomics) to build the predict model, and a high prediction accuracy (test accuracy = 0.87) on the independent test data at treatment fraction of 5 was achieved for predicting acute skin toxicity at the end of RT (12, 13). However, the prediction performance is not sufficient enough to be as an effective clinical decision support tool for intervention and management of dermatitis in breast cancer patients, probably due to the 2-D surface imaging with limited information provided rather than 3-D volume imaging with one more dimension information offered. The models built on 2-D surface thermal imaging constrain their usage for 3-D dose distribution optimization guidance. Furthermore, the extra usage of thermal imaging devices and additional procedures involved might increase the labor burdens in the breast radiation oncology clinic and reduce the patient throughput.

In this study, we investigated 3-D planning CT volume imaging and machine learning frameworks to develop a quantitative prediction tool for radiation-induced acute radiodermatitis in breast cancer patients before RT treatment. This multicenter retrospective study was performed using a novel 3-D dose-gradient-based multi-region radiomics technique with the data encapsulation screening method integrated. The gradient boosting decision tree (GBDT) algorithm was used to build the predictive model. We hypothesized that acute radiodermatitis is associated with the 3-D region-based characteristic radiomics signatures in breast cancer patients before RT.

## 2 Methods and materials

### 2.1 Patients and CT scans

This study retrospectively reviewed 256 patients with stage 0-IV breast cancer, who underwent post-surgery (i.e., lumpectomy, mastectomy, or breast reconstruction) intensity-modulated radiation therapy or volumetric modulated arc therapy RT with or without concurrent chemotherapy and/or Hormone therapy, at 3 cancer centers including our hospital from October 2018 to August 2021 under institutional review board approval. The patients received a prescription dose of whole breast and/or chest wall irradiation mainly using regimens of 50 Gy in 25 fractions or 42.5 Gy in 16 fractions with an optional boost of 10 Gy in 5 fractions to the tumor bed using the 6 MV photons. The patients were monitored for skin symptoms from the start of RT to at least 1 month after the completion of RT. A total of 214 patients (144 patients with ≥ 2 grade skin toxicity) were selected based on the exclusion criteria including (1) prior or subsequent RT to the chest, (2) previous skin disorder, (3) with dose boost using electron therapy, (4) male patients, (5) loss of clinical characteristics records. Informed consent from all the patients was obtained before the study. All study participates were graded for skin toxicity using Radiotherapy Oncology Group (RTOG), Common Terminology Criteria for Adverse Events (CTCAE) Ver. 4 (6, 7).

In our study, all patients underwent breathing training before radiotherapy; 88 of them with left-sided breast cancer were treated with deep inhalation breath-hold (DIBH) radiotherapy technique, and their CT scans were completed in breath-hold state. Other 126 of them with right-sided breast cancer underwent 4D-CT scans in free-breathing state. CT scans of the patients for treatment planning were mainly conducted using a Philips Brilliance Big Bore CT (Philips Medical Systems, Cleveland, OH, USA) 2 to 7 days before RT. The imaging parameters of the CT scans include voltage (120 kVp), tube current (325 mA or 375mA), exposure time (800 ms or 933 ms), pixel size (0.5×0.5 mm or 0.6×0.6 mm), slice thickness (5 mm), and image size (XY: 768×1024, Z: around 80). The Pinnacle (Philips Medical Systems, Andover, MA) or Eclipse treatment planning systems (Varian Medical Systems, Palo Alto, CA) were used for the calculation of the radiation dose distribution of contoured treatment volumes.

**Figure 1:**
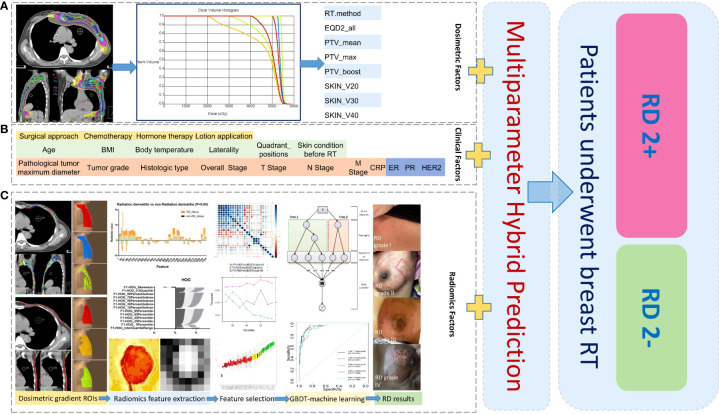
Schematic diagram of data analysis for machine learning in this study: Collection and analysis based on dosimetric factors **(A)**, patient clinical factors **(B)**, and radiomics factors **(C)** extracted from different dose-gradient regions of patients. ROIs, regions of interest; RD 2+, radiodermatitis with ≥ 2 grade; RD 2-, radiodermatitis with< 2 grade.

The planning CT scans and associated dose distributions of eligible patients were collected for data analysis and model building (**Figure 1**). Clinical characteristics of the patients include age, body mass index (BMI), body temperature, tumor laterality, tumor quadrant positions, pathological tumor size (e.g., tumor maximum diameter), tumor grade, tumor histology type, TNM stage, overall stage, CRP, ER, PR, HER-2, surgery method, chemotherapy, hormone therapy, etc. (**Table 1**).

**Table 1:** Demographic and clinical information of the patients (n=214) in the study.

Demographic and clinical characteristics		Grade ≤ 1(n = 70)	Grade ≥ 2(n = 144)	P value (Chi-squared/MUW test)
Age (mean (SD))		50.04 (9.44)	49.48 (9.62)	0.804
BMI (mean (SD))		22.94 (3.03)	23.25 (2.70)	0.773
Body temperature (mean (SD))		36.67 (0.33)	36.70 (0.35)	0.692
Laterality (%)	Left	44 (62.9)	82 (56.9)	0.411
	Right	26 (37.1)	62 (43.1)	
Quadrant position (%)	Upper-Outer	17 (24.3)	23 (15.9)	0.207
	Upper-Inner	26 (37.1)	57 (39.6)	
	Lower-Outer	13 (18.6)	54 (37.5)	
	Lower-Inner	14 (20.0)	10 (6.9)	
Tumor maximum diameter (cm) (mean SD)		1.97 (0.91)	2.28 (1.68)	0.759
Tumor grade (%)	I	5 (7.2)	13 (9.0)	0.958
	II	36 (51.4)	69 (47.9)	
	III	29 (41.4)	62 (43.1)	
Histologic type (%)	DCIS	10 (14.3)	13 (9.0)	0.114
	IDC	59 (84.3)	124 (86.1)	
	ILC	1 (1.4)	5 (3.5)	
	IMC	0 (0.0)	1 (0.7)	
	LCIS/DCIS	0 (0.0)	1 (0.7)	
Overall Stage (%)	0	4 (5.7)	9 (6.3)	0.189
	I	14 (20.0)	16 (11.1)	
	IIA	17 (24.3)	31 (21.5)	
	IIB	0 (0.0)	1 (0.7)	
	IIIA	3 (4.3)	12 (8.3)	
	IIIB	18 (25.7)	30 (20.8)	
	IIIC	13 (18.6)	40 (27.8)	
	IV	1 (1.4)	5 (3.5)	
T Stage (%)	Tx~is	1 (1.4)	4 (2.8)	0.109
	T0	3 (4.3)	5 (3.4)	
	T1	4 (5.7)	5 (3.5)	
	T2	43 (61.4)	74 (51.4)	
	T3	17 (24.3)	48 (33.3)	
	T4	2 (2.9)	8 (5.6)	
N Stage (%)	0	36 (51.4)	66 (45.8)	0.595
	1	18 (25.7)	46 (31.9)	
	2	11 (15.7)	21 (14.6)	
	3	5 (7.2)	11 (7.7)	
M Stage (%)	0	70 (100.0)	143 (99.3)	0.002
	1	0 (0.0)	1 (0.7)	
CRP (mg/l) (mean (SD))		2.27 (4.19)	2.36 (5.03)	0.876
ER (%)	Positive	55 (78.6)	114 (79.2)	0.960
	Negative	15 (21.4)	30 (20.8)	
PR (%)	Positive	50 (71.4)	112 (77.8)	0.312
	Negative	20 (28.6)	32 (22.2)	
HER2 (%)	Positive	14 (20.0)	34 (23.6)	0.554
	Negative	56 (80.0)	110 (76.4)	
Surgery method (%)	Lumpectomy	41 (58.6)	84 (58.3)	0.928
	Mastectomy	27 (38.6)	59 (41.0)	
	Reconstruction	2 (2.8)	1 (0.7)	
Chemotherapy (%)	No	13 (18.6)	32 (22.2)	0.541
	Yes	57 (81.4)	112 (77.8)	
Hormone therapy (%)	No	44 (62.9)	77 (53.5)	0.195
	Yes	26 (37.1)	67 (46.5)	
EQD2_all (mean (SD))		52.16 (5.18)	52.66 (4.16)	0.466
Lotion application (%)	No	6 (8.6)	5 (3.5)	0.115
	Yes	64 (91.4)	139 (96.5)	
PTV_mean (mean (SD))		5098.71 (325.99)	5139.06 (317.50)	0.838
PTV_max (mean (SD))		5773.97 (477.05)	5839.24 (454.27)	0.937
PTV_boost (%)	Yes	41(58.6)	80(55.6)	0.678
	No	29(41.4)	64(44.4)	
SKIN_mean (mean (SD))		3608.40 (493.26)	3587.22 (570.25)	0.730
SKIN_max (mean (SD))		5447.94 (489.98)	5535.82 (464.63)	0.504
SKIN_V20 (mean (SD))		87.26 (10.00)	85.65 (12.01)	0.316
SKIN_V30 (mean (SD))		70.60 (14.74)	69.69 (15.31)	0.461
SKIN_V40 (mean (SD))		44.59 (21.24)	43.83 (20.93)	0.626

SD, standard deviation; BMI, body mass index; DCIS, ductal carcinoma in situ; IDC, invasive ductal carcinoma; ILC, invasive lobular carcinoma; IMC, invasive mammary carcinoma; LCIS, lobular carcinoma in situ; HER2, human epidermal growth factor receptor 2; ER, estrogen receptor; PR, progesterone receptor; EQD2_all,equivalent dose of all treatment phase at 2Gy/fraction.

All patients were informed by nurses about the basic skin cares before treatment, including daily rinsing of the breast skin surface with warm water, keeping the breast skin moist and clean, and avoiding friction of the skin of breasts by hard clothing. If the patients are prone to RD 2+, they may be advised to use silver sulfadiazine 1% three times per day for 5 weeks. All the patients and family members confirmed the consensus of cooperation.

### 2.2 Data processing and model building

#### 2.2.1 Radiomics feature extraction

The construction and application of a radiation dermatitis prediction model was illustrated in **Figure 1**. A total of 884 radiomics features were extracted from each delineated ROI by using the open-source image biomarker explorer (IBEX) software platform (14). The radiomics features extracted includes seven categories: shape, intensity direct, intensity histogram, gray-level co-occurrence matrix (GLCM) (2.5D), neighbor intensity difference (2.5D), gray level run length matrix (2.5D), and intensity histogram Gaussian fit. Radiomics features were extracted from PTV regions defined with 100%, 105%, 108% of the prescribed dose and skin regions defined with 20-Gy, 30-Gy, 40-Gy isodose of the skin for the following model building.

#### 2.2.2 Null interpolation

Based on the fact that missing of clinical and dosimetric variable values are types of data missing completely at random (MCAR) or missing at random (MAR), two methods of maximum likelihood (ML) and multiple imputation (MI) can be used to fill null variable values. We used the ML method to impute the linear null data; the MI method was applied to fill the non-linear null data. For radiomics features, since the proportion of null data is very low (<10%) and the correlation between feature variables is high, the method of directly removing null data should not generate the biased estimation.

#### 2.2.3 Unbalanced data handling

Training on imbalanced dataset would create a biased prediction in the minority class of dataset. The degree of imbalance of dataset is based on the proportion of a minority class in the whole dataset and could range from mild (20-40%), moderate (1-20%) to extreme (<1%) imbalances (15). Previous studies showed that resampling approach is a useful pre-processing step to handle the imbalanced dataset (16, 17). This method modifies the imbalance distribution of the majority and minority classes at the data level before training with classifiers. In this study, due to a mild imbalanced dataset used (non-RD2+ patients/total patients =32.7%), an imbalanced adjustment strategy of Synthetic Minority Oversampling Technique (SMOTE) was utilized before all the data sets were trained. SMOTE is a very popular algorithm for oversampling of the minor class data. Briefly, SMOTE takes k data from k-NN (near neighbors) for each data in the minor class to perform oversampling, and then generates new data by obtaining “in-line” data with one of the randomly chosen k-NN data results for a number of magnification.

#### 2.2.4 Screening of prediction variable

The p values were calculated for clinical and dosimetric variables (**Table 1**), in which the chi-square test was used by default for categorical variables, and the MUW test was used by default for continuous variables. If the data did not meet the conditions for the chi-square test, the fisher’s exact test was used instead. The variables with P value< 0.5 were selected for multiple-variable logistic regression analysis in the following step. Because the sample size of this study is relatively not large, the current data might not represent the actual situation, and the low p value might cause missing of important variables that account for the prediction model. In performing multiple-variable logistic regression of clinical and dosimetric variables, we set a relatively high P value of 0.5 (compared to P< 0.1 or P< 0.05) to avoid too few variables included in the regression analysis, which may loss valuable variables for further analysis. This resulted in 8 variables included in the regression equation (**Table 2**).

**Table 2:** Multiple-variable logistic regression of selected clinical and dosimetric variables.

Variable	Coefficient	Wald Z	Pr (>|Z|)
Laterality	0.6983	1.86	0.0628
Quadrant position	0.1183	2.09	0.0362
Histologic type	0.2709	0.68	0.4967
T Stage	0.1641	0.69	0.4923
PR	-0.2727	-0.73	0.4631
Hormone therapy	0.4601	1.35	0.1776
EQD2_all	-0.1483	-0.75	0.4525
Lotion application	0.7188	1.08	0.2789

For the radiomics data extracted from the 6 ROIs, the MWU test was firstly performed with P value < 0.05 set, and then redundant features with variance ≤ 0.05 were deleted. In the next step, the pairwise correlation coefficient between one variable and all the remaining variables was calculated, and variables with correlation coefficient ≥ 0.9 were deleted. When the correlation coefficients of two variables are the same, the variable with the larger correlation with the classification result was kept. Meanwhile, a variance inflation factor (VIF) was calculated for multiple linear tests on the remaining variables, in which all variables with VIF ≥ 10 were removed. Then, a decision tree encapsulation screening method was applied to filter the variables for the following prediction model building. The encapsulation screening method integrated the feature selection process with the training process, and used the predictive ability of the model as a measure of feature selection to select a high-quality subset of variables.

#### 2.2.5 Model training and validation

The GBDT machine learning algorithm was used to train and validate the clinical and dosimetric, radiomics, and combined prediction models, respectively. Gradient boosting is an integrated boosting method, which iterates the new learner through the gradient descent algorithm, and boosting refers to connecting multiple weak learners in series to generate a new strong learner.

For binary GBDT in this study, the loss function is defined as (18)


(1) L(y,f(x))=log(1+exp(−yf(x)))

where *y* is the label, and *f*(*x*) denotes the prediction value. Then the negative gradient error at the current time is defined as


(2) $$r_{ti}=-{\left[\frac{\partial L(y,f(x))}{\partial f(x)}\right]}_{f(x)=f_{t-1}(x)}=\frac{y_{i}}{1+\exp(y_{i}f(x_{i}))}$$

For the generated decision tree, the best residual fitting value of each leaf node is


(3) $$c_{tj}=\arg\min\sum_{x_{i}\in R_{tj}}(log(1+exp(y_{i}f_{t-1}(x_{i}+c))))$$

Since the above equation is difficult to be optimized in a computer, we use the following loss function to approximate it instead:


(4) $$c_{tj}=\frac{\sum_{x_{i}\in R_{tj}r_{ti}}}{\sum_{x_{i}\in R_{tj}}|r_{ti}|*(1-|r_{ti}|)}$$

The pseudocode of the binary GBDT is as follows:

Algorithm Gradient Boosting Trees Algorithm
1 Initialize 
$f_{0}(x)=\arg\min_{\gamma }\sum_{i=1}^{N}L(y_{i},\gamma )$
2. For m=1 to M:
(a) For i=1,2,…,N compute: 
$r_{im}=-{\left[\frac{\partial L(y_{i},(f(x_{i}))}{\partial f(x_{i})}\right]}_{f=f_{m-1}}$
(b) Fit a regression tree to the targets r_im_ gibing terminal regions,
R_jm_, j = 1,2,…,Jm compute:
(c) For j = 1,2,…,Jm compute:*γ*_*jm*
_=*argmin*_*γ*
_∑_*x*_*i*
__∈__*R*
__*jm*
_
_*L*(*y*_*i*
_,*f*_*m*−1_(*x*_*i*
_)+*γ*)
(d) Update 
$f_{m}(x)=f_{m-1}(x)+\sum_{j=1}^{Jm}\gamma_{jm}I(x_{i}\in R_{jm})$
3. Output. 
$\hat{f}(x)=f_{M}(x)$

The entire data set was divided into 5 equal sub-folds with the ratio of close to 1:1 for RD 2+ and non-RD 2+ patients in each sub-fold, and the patients in each sub-fold do not appear repeatedly. 70% of the data in each sub-fold were used for GBDT model training, and the remaining 30% were used for validation. A *gbm* package in *Rstudio* was used to implement the GBDT algorithm (19). Since the problem is a classification problem, the Bernoulli distribution was selected in the loss function. The learning rate shrinkage parameter was set at 0.05, and the number of decision tree was set to 10000. The optimal number of iterations and the importance of each explanatory variable were determined by using a 5-fold cross-validation.

## 3 Results

### 3.1 Variable selection and data handling

With the null imputation method being applied to the clinical and dosimetric datasets, total of 29 clinical and dosimetric variables were retained for further analysis. The number of remained non-null radiomics features extracted from the PTV_100PD, PTV_105PD, PTV_108PD, SKIN_20Gy, SKIN_30Gy, and SKIN_40Gy were 812, 789, 674, 684, 657, and 664, respectively.

After the SMOTE method was applied, the total number of samples was increased from 214 to 280, and the number of non-RD 2+ cases was increased from 70 to 140. In the new balanced data, the ratio of RD 2+ and non-RD 2+ patients was close to 1:1.

### 3.2 Model training and validation

As mentioned above, the 8 clinical and dosimetric variables selected were fed into the GBDT model for training. The performance of GBDT model in the training and validation datasets using the selected clinical and dosimetric variables is shown in **Table 3**. It is observed that the clinical and dosimetric characteristics showed moderate predictive power for RD 2+, even in the best performance in the second and third sub-folds in the training and validation set (i.e., AUC of 0.839 with 95% CI of 0.788-0.891, and AUC of 0.816 with 95% CI of 0.705-0.927).

**Table 3:** The GBDT model performance in training and validation dataset using selected clinical and dosimetric variables. The bold values indicate the best prediction performance in the training set and validation set, respectively.

Type-GBDT		Folds	1-foldsModel-1	2-foldsModel-2	3-foldsModel-3	4-foldsModel-4	5-foldsModel-5
**Training-set**	RD 2+/Non-RD 2+		0.830 95% CI: 0.777-0.884 (DeLong)	**0.839** 95% CI: 0.788-0.891(DeLong)	0.786 95% CI: 0.727-0.845 (DeLong)	0.802 95% CI: 0.744-0.861 (DeLong)	0.811 95% CI: 0.754-0.867(DeLong)
**Validation-set**	RD 2+/Non-RD 2+		0.725 95% CI: 0.587-0.863(DeLong)	0.743 95% CI: 0.611-0.877 (DeLong)	**0.816** 95% CI: 0.705-0.927 (DeLong)	0.748 95% CI: 0.618-0.879(DeLong)	0.759 95% CI: 0.631-0.886(DeLong)

With the MWU test, zero-variance test, correlation test, VIF verification and tree encapsulation screening method being successively applied to the radiomics dataset, we obtained 20 radiomics features from the 2 types of ROIs with 6 dose levels. The VIFs of these radiomics features and their AUCs in predicting RD 2+ were shown in **Table 4**. As can be observed from the table, these radiomics features showed limited prediction performance on their own, such as PTV_100PD_radiomics_average (AUC, 0.566 [95% CI: 0.497-0.632]), SKIN_20Gy _radiomics_average (AUC, 0.569 [95% CI: 0.501-0.636]), and so on.

**Table 4:** AUC of 20 radiomics features after variable screening using decision tree encapsulation screening method. The bold values indicate the average values across the dose regions.

Feature	VIF	AUC	95%CI(DeLong)
PTV_100Pd_F2.GLCM25270.7_Corr	8.608	0.591	0.524-0.658
PTV_100Pd_F4.ID_LocalStdMedian	8.945	0.604	0.537-0.670
PTV_100PD_F4.ID_Range	1.407	0.510	0.441-0.577
PTV_100PD_F8.ShapeMax3Ddiameter	1.551	0.544	0.476-0.612
PTV_100PD_F6.IHGaussFit1GaussMean	1.311	0.576	0.507-0.644
**PTV_100PD_radiomics_average**	**4.364**	**0.566**	**0.497-0.632**
PTV_105PD_F2.GLCM25.333.7_Corr	1.208	0.592	0.525-0.659
PTV_105PD_F4.ID_LocalEntropyMax	1.080	0.570	0.502-0.637
PTV_105PD_F8.ShapeMeanBreadth	1.123	0.587	0.520-0.655
**PTV_105PD_radiomics_average**	**1.137**	**0.583**	**0.516-0.650**
PTV_108PD_F1.GOH0.975Quantile	1.277	0.588	0.519-0.656
PTV_108PD_F2.GLCM25180.1Dissimilarity	1.831	0.574	0.506-0.642
PTV_108 PD_F8.ShapeNumberOfObjects	1.292	0.606	0.540-0.672
PTV_108PD_F2.GLCM2590.7_IV	1.301	0.568	0.500-0.636
**PTV_108PD_radiomics_average**	**1.425**	**0.584**	**0.516-0.652**
SKIN_20Gy.F2.GLCM25225.4Contrast	3.749	0.570	0.503-0.637
SKIN_20Gy.F8.ShapeConvexHullVolume3D	1.827	0.554	0.486-0.622
SKIN_20Gy.F8.ShapeMeanBreadth	7.401	0.582	0.515-0.650
**SKIN_20Gy _radiomics_average**	**4.326**	**0.569**	**0.501-0.636**
SKIN_30Gy_F2.GLCM25225.4Contrast	1.411	0.577	0.510-0.645
SKIN_30Gy_F4.ID_LocalRangeMax	1.286	0.613	0.546-0.680
SKIN_30Gy_F6.IHGaussFit1GaussStd	1.255	0.641	0.576-0.706
SKIN_30Gy_F1.GOH_MAD	1.351	0.591	0.524-0.658
SKIN_30Gy_F8.ShapeMax3DDiameter	1.075	0.655	0.59040.719
**SKIN_30Gy _radiomics_average**	**1.276**	**0.615**	**0.549-0.682**

As can be observed in **Table 5**, using combined radiomics features from all the ROIs, the prediction was improved significantly for the GBDT model both in training and validation sub-folds (e.g., AUC of 0.998 [95% CI, 0.996-1] for the training set, AUC of 0.907 [95% CI, 0.829-0.985] for the validation set).

**Table 5:** The GBDT model performance in training and validation dataset using 20 selected radiomics features. The bold values indicate the best prediction performance in the training set and validation set, respectively.

Type-GBDT		Folds	1-foldsModel-1	2-foldsModel-2	3-foldsModel-3	4-foldsModel-4	5-foldsModel-5
**Training-set**	RD 2+/Non-RD 2+		0.998 95% CI: 0.996-1.0 (DeLong)	0.997 95% CI: 0.993-1.0 (DeLong)	0.997 95% CI: 0.994-1.0 (DeLong)	0.974 95% CI: 0.954-0.993 (DeLong)	**0.998** 95% CI: 0.996-1.0 (DeLong)
**Validation-set**	RD 2+/Non-RD 2+		0.881 95% CI: 0.782-0.981 (DeLong)	**0.907** 95% CI: 0.829-0.985 (DeLong)	0.901 95% CI: 0.814-0.987 (DeLong)	0.867 95% CI: 0.777-0.958 (DeLong)	0.875 95% CI: 0.769-0.980 (DeLong)

**Figure 2:**
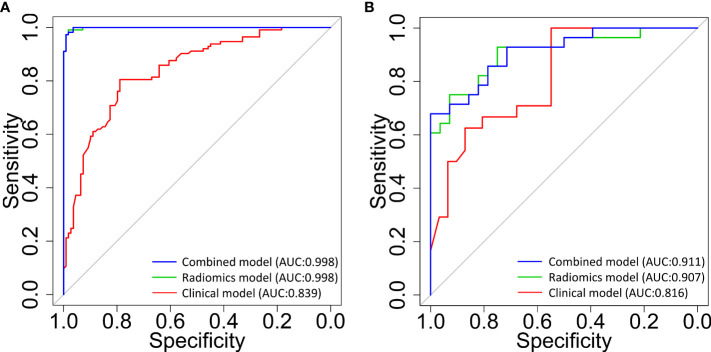
The receiver operating characteristic (ROC) curves for the classification of patients with and without radiodermatitis (RD 2+). The 3 curves are for classifiers that were built using clinical and dosimetric (red line), radiomics signatures within multiple ROIs (green line), and the combination of clinical, dosimetric, and radiomics features within multiple ROIs (blue line), respectively. **(A)**: prediction model performance in the training set; **(B)**: prediction model performance in the validation set. AUC, area under the curve; ROIs, regions of interest.

As shown in **Table 6**, in the GBDT model built on the combined clinical, dosimetric and radiomics characteristics, the best performance of the model resided in the first and fourth sub-fold in the training and validation set, with a AUC of 0.998 [95% CI:0.996-1.0] and a AUC of 0.911 [95% CI: 0.838-0.983], respectively. The best performance with the highest AUC value of each sub-folds in training and validation set of the three GBDT models were summarized in **Figure 2**.

**Table 6:** The GBDT model performance in training and validation dataset using selected radiomics combined with clinical and dosimetric variables. The bold values indicate the best prediction performance in the training set and validation set, respectively.

Type-GBDT	Folds	1-foldsModel-1	2-foldsModel-2	3-foldsModel-3	4-foldsModel-4	5-foldsModel-5
**Training-set**	RD 2+/Non-RD 2+	**0.998** 0.996-1.0 (DeLong)	0.996 0.991-1.0 (DeLong)	0.998 0.991-1.0 (DeLong)	0.996 0.991-1.0 (DeLong)	**0.983** 0.970-0.995 (DeLong)
**validation-set**	RD 2+/Non-RD 2+	0.857 0.755-0.960 (DeLong)	0.908 0.835-0.982 (DeLong)	0.816 0.706-0.927 (DeLong)	**0.911** 0.838-0.983 (DeLong)	0.837 0.723-0.950 (DeLong)

### 3.3 Important predictor analysis

**Figure 3:**
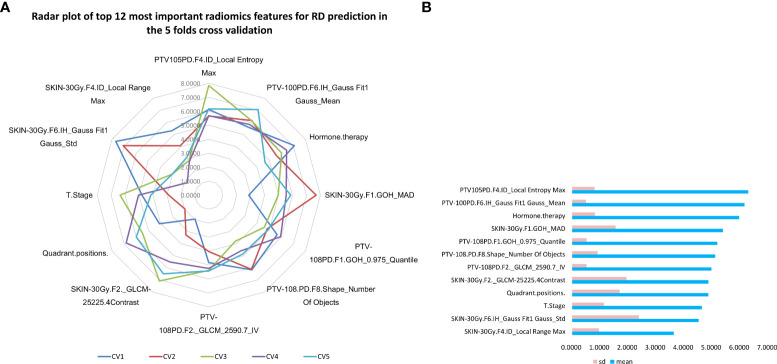
Top 12 most important variables in the combined GBDT model for radiodermatitis prediction. **(A)** the radar plot of top 12 most important prediction features in 5 folds cross validation GBDT machine learning process; **(B)** The mean and standard deviation of importance measures of the top 12 most important radiodermatitis prediction features sorted by the average measures.

Meanwhile, the top 12 most important characteristics as well as their corresponding importance measures (i.e., mean and standard deviation) for RD 2+ prediction in the combined GBDT model were shown in **Figure 3**. Three clinical characteristics were selected in this top variable list, including Hormone.therapy, T.Stage, and Quadrant.positions. Four radiomics features from the SKIN_30Gy region, including ID_Local Range Max, IH_Gauss Fit1 Gauss_Std, GOH_MAD and GLCM-25225.4Contrast, were identified as important features for prediction of RD 2+. Five radiomics features, including GLCM_2590.7_IV, Shape_Number Of Objects and GOH_0.975_Quantile from PTV_108PD, IH_Gauss Fit1 Gauss_Mean and ID_Local Entropy Max from PTV_105PD, were chosen in this top list. Most of these features focus on describing the region heterogeneity and complexity of the textures in patients’ PTV and skin volumes.

**Figure 4:**
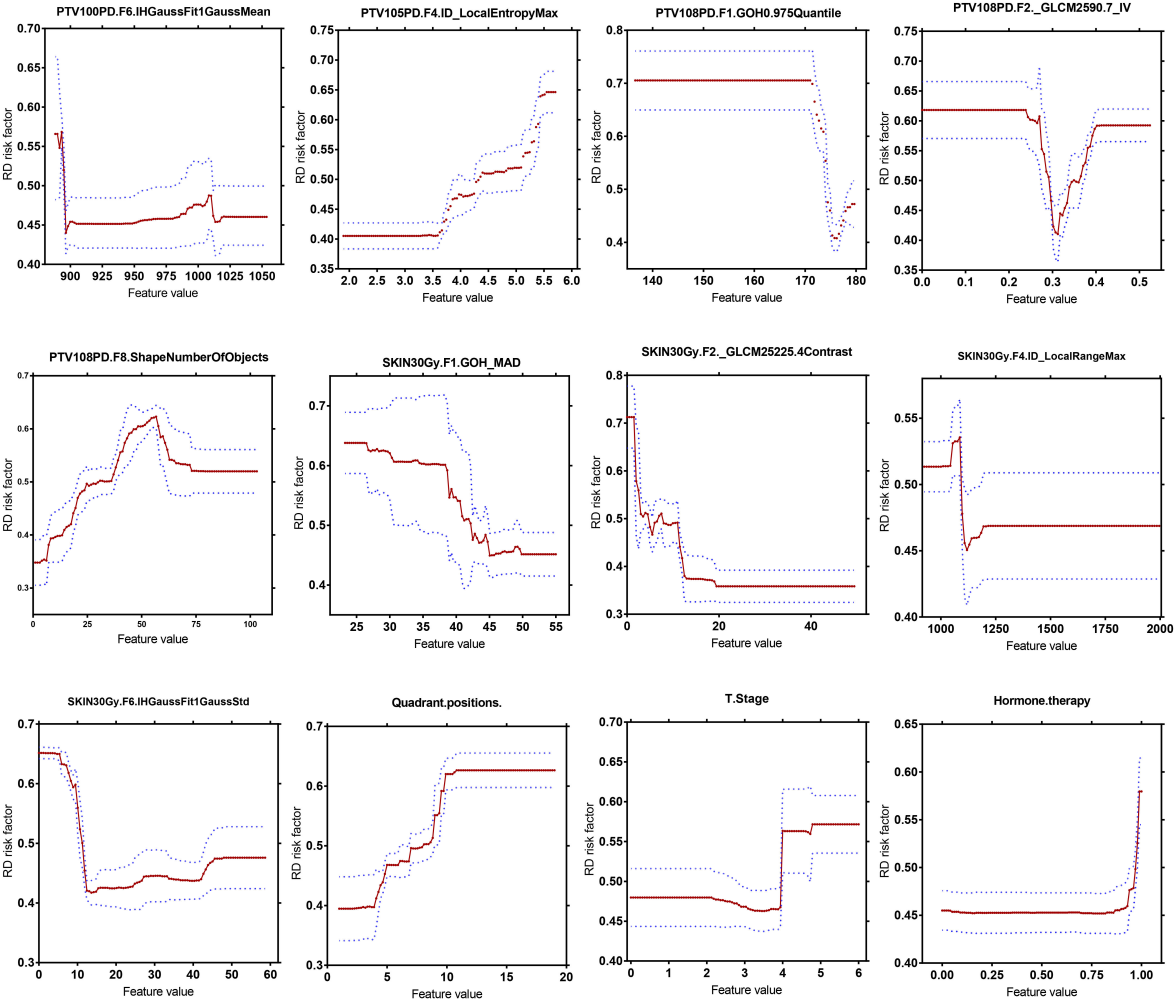
Quantitative correlation analysis of changes in top 12 most important variables in the GBDT model with changes in risk scores of radiodermatitis.

**Figure 5:**
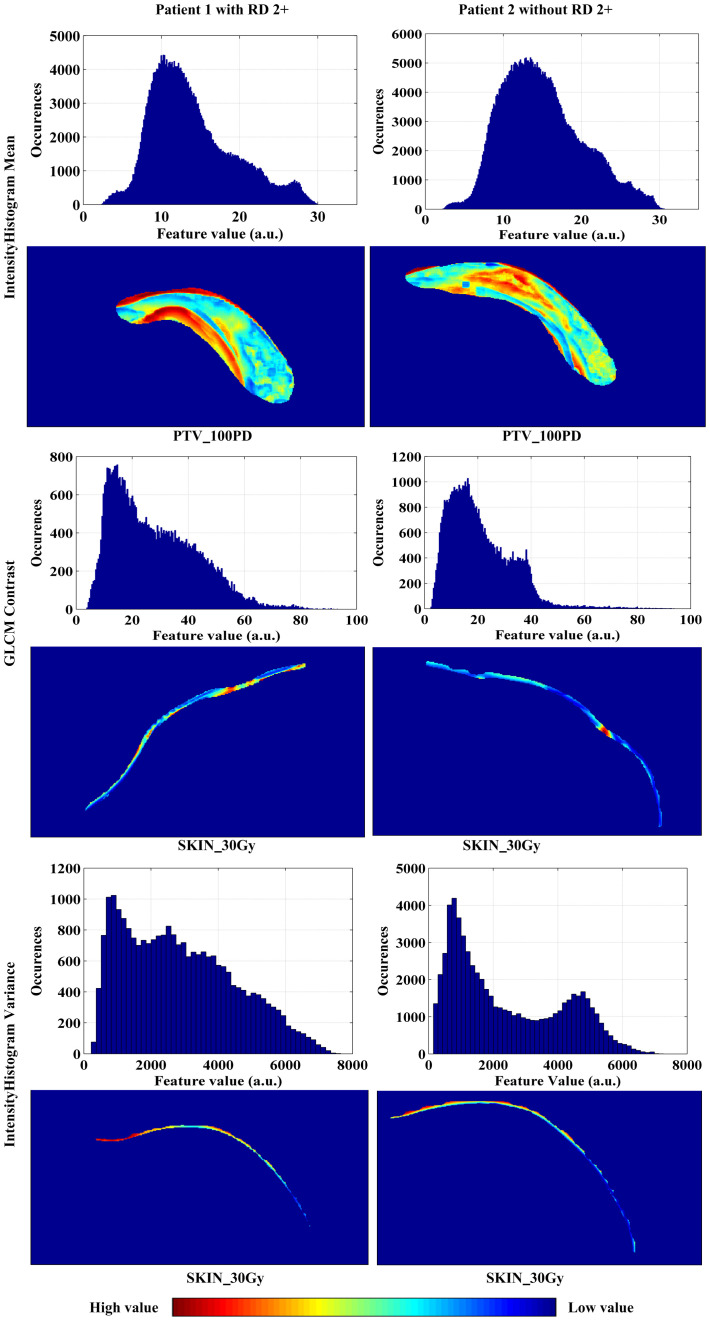
The amplitude and spatial distributions of the feature values of IH_Gauss Fit1 Gauss Mean, GLCM_25225.4Contrast, and IH_Gauss Fit1 Gauss_Std extracted from the sub-volumes within the ROIs of PTV_100PD, SKIN_30Gy, SKIN_30Gy, respectively, for patients with and without RD 2+.

As illustrated in **Figure 4**, changes of the top 12 variable values were correlated with risk scores of RD 2+. For instance, the increase of SKIN_30Gy.GLCM-25225.4Contrast value was correlated to the decreased risk score for the occurrence of RD 2+; and it seems like that a threshold of SKIN_30Gy.IH_Gauss Fit1 Gauss_Std can be set to identify the patients with a high risk for RD 2+. We further explored the distributions (i.e., spatial and amplitude) of feature values, calculated from sliding sub-volumes (e.g., containing 7×7×7 voxels) within the ROIs, of several variables in the top list. **Figure 5** shows the exemplary amplitude and spatial distributions of the feature values of IH_Gauss Fit1 Gauss Mean, GLCM_25225.4Contrast, and IH_Gauss Fit1 Gauss_Std extracted from the sub-volumes within the ROIs of PTV_100PD, SKIN_30Gy, SKIN_30Gy, respectively, for patients with and without RD 2+.

## 4 Discussion

There is currently no gold standard for the prevention and management of RD 2+ for breast cancer patients. Many interventions are based on the experience of physicians and nurses, anecdotal evidence, or low-level evidence, and there are very limited prospective data to guide interventions currently. The goal of treatment is primarily to improve patient comfort, minimize the risk of further damages, and promote wound healing. This study aimed to provide an innovative method to quantitatively assess the risk of radiation dermatitis before treatment, which will greatly reduce the clinical cost of trial and error for high-risk patients, and offer the opportunity to optimize the radiotherapy plan for high-risk patients just before treatment.

Ionizing radiation essentially damages the mitotic ability of clonogenic or stem cells within the basal layer of epidermis, thus preventing the process of repopulation and weakening the integrity of the skin. The degrees of damage range from mild to severe as telangiectasias, erythema, desquamation, keratinocyte cell death, fibrosis and inflammatory response (10). The incidence of grade 2 or higher radiation dermatitis in this study (approximately 67.3%) was similar to that in previous studies (31%-50%) (20). In this study, we extracted radiomics features from skin- and PTV-related ROIs defined by different dose gradients in the planning CT images. It was found that these radiomics characteristics combined with clinical and dosimetric factors significantly improved the predictive accuracy of RD 2+. The results showed the potential of taking the risk of RD 2+ and the radiation sensitivity of multiple ROIs into account in the RT planning procedures, which facilitates personalized radiation dose distribution at the planning stage of RT to improve outcomes for patients at the high risk of RD 2+.

In this study, all patients were divided into three groups: (1) lumpectomy (i.e., partial breast resection surgery or breast conserving surgery) group, (2) mastectomy group, (3) breast reconstruction group. Previous studies found that lumpectomy was associated with a higher rate of moderate or severe dermatitis than mastectomy (63% vs. 24%, P = 0.003) (21–23), which might be due to the local dose escalation after breast conserving surgery. However, our data did not show the same situation. In the lumpectomy cohort, RD 2+ was found in 80 (66.1%) out of 121 patients who underwent a dose escalation to the tumor bed. In the mastectomy cohort, 68.6% (59/86) patients developed RD 2+. There was no significant statistical difference between the two groups (p = 0.556), which suggested that local increase of radiation dose might not be an important risk factor for RD 2+. Meanwhile, it was found that there was no significant difference in the occurrence probability of RD 2+ between lumpectomy and mastectomy groups (p=0.441), which indicated that the surgery method might not be a risk factor for RD 2+.

Previous study demonstrated that higher biologically equivalent dose was correlated to an increase in the rate of moderate or severe dermatitis (12). Our results showed that there were no statistically significant differences in EQD2_all (P = 0.457) between patients with and without RD 2+ by using the MUW test. Patient large breast size and high BMI have been found to be independent risk factors of acute skin toxicity, including moist desquamation (24). A greater self-bolusing effect is supposed to increase toxicity in the inframammary and axillary folds, due to the dose buildup of skin-on-skin. Therefore, patients with large breast size and/or high BMIs are prone to RD 2+ due to the greatest areas of skin-on-skin overlap. However, our results showed that the BMI, as well as chemotherapy, expression of hormone receptors or HER2, were not directly associated with RD 2+, which was consistent with the similar study carried by a French study team (13).

Although the clinical and dosimetric characteristics were not significantly predictive of symptomatic RD 2+ in multivariable logistic modeling, they showed good performance both in the training and validation datasets when the GBDT algorithm was adopted (e.g., best AUCs in 5-flod CV in training and validation dataset are 0.839 with 95% CI of 0.788-0.891 and 0.816 with 95% CI of 0.705-0.927, respectively) (**Table 3**). This suggested that the GBDT algorithm was the appropriate choice for the problem in this study.

By using decision tree encapsulation screening method, we screened out 5, 3, 4, 3, and 5 features from the 5 ROIs of PTV_100PD, PTV_105PD, PTV_108PD, SKIN_20Gy, and SKIN_30Gy, respectively. The number of radiomics features retained from the PTV ROIs was greater than the skin ROIs. The predictive ability of radiomics features of a single ROI was relatively low, which indicated that it was difficult to extract predictors with excellent prediction performance from a single ROI. However, when we used all the screened 20 radiomics features from multiple ROIs, the best AUC values of the prediction model reached 0.998 with 95% CI of 0.996-1.0 and 0.907 with 95% CI of 0.829-0.985 in the training and validation set, respectively. Therefore, we speculate that the occurrence of RD 2+ is not only directly related to the patient’s skin, but also the characteristics of the PTV adjacent to the skin which will also have an important impact on the occurrence of RD 2+.

In this study, our analysis found that RD 2+ was not strongly correlated to the dose characteristics of the skin as well as those of PTV adjacent to the skin, whereas the radiomics indicators of PTV_100PD, PTV_105PD, PTV_108PD, SKIN_20Gy, and SKIN_30Gy showed strongly correlated to the occurrence of RD 2+. This suggested that radiomics characteristics of these ROIs of the skin and PTV play more important role in the prediction of RD 2+ than the dosimetric characteristics for breast cancer patients treated with RT. For the sake of safety, driving those PTV and skin regions to the low-abundance regions of RD 2+-sensitive radiomics features holds the potential to reduce the occurrence of RD 2+.

In the combined prediction model, radiomics features extracted from the SKIN_30Gy, PTV_100PD, PTV_105PD, and PTV_108PD were the most important predictors of RD2+; while clinical characteristics, including estrogen therapy, tumor T stage, and tumor quadrant positions, were also important predictors. A previous study reported the volume of skin receiving a dose >35 Gy (SKIN_V35), PTV-V100%, PTV-V105%, PTV-V107% (i.e., volumes receiving percentage of prescribed dose within PTV) were the most significant dosimetric predictors associated with >50% probability of RD 2+ toxicity (20). Although our results did not show the strong correlation between the volumes of SKIN_V30 and/or SKIN_V40 and the occurrence of RD 2+, and the correlations between the volumes of PTV-V100%, PTV-V105%, and/or PTV-V107% and the occurrence of RD 2+ were not analyzed, our results revealed strong correlations between specific radiomics features extracted from these volumes and the occurrence of RD 2+.

As can be found from **Tables 5**, **6** and **Figure 2**, the model performance was not improved significantly when the clinical and dosimetric characteristics were added for training. This fact highlighted the role of radiomics features, extracted from the multiple dose-gradient-based ROIs of planning CT images of the patients, in the prediction of RD 2+ before treatment using the GBDT modeling method. This can be very helpful if clinical and/or dosimetric details of the patients were lost, as collecting these data is a labor intensive and time consuming task in practice.

The reason why we chose CT images for radiomics study rather than MRI images is that planning CT images were obtained within a week before the start of RT, whereas MRI images were usually acquired at the beginning of patient admission. As such, the patients’ CT images reflect the baseline of the skin condition before RT more than MRI images do. Although MRI has advantages over CT in breast imaging, Wang et al. conducted a predictive model for the fibrotic level of neck muscles after radiotherapy by using radiomic features extracted from the MRI images before and after radiotherapy and planning CT in nasopharyngeal carcinoma patients, and they found that the prediction model based on CT radiomics features has better performance in the prediction of the grade of post-radiotherapy neck fibrosis (25). Therefore, we adopted extraction of radiomics features from patients’ CT images instead of MRI images, which are usually not available due to the high cost.

The robustness of radiomics features was usually influenced by respiratory motion (26). For the patients with breast cancer, the respiratory motion was mainly manifested in the anterior-posterior direction. In our study, the left-side breast cancer patients underwent CT scans in the breath-holding state, therefore, the CT radiomics features from these patients was relatively reliable. For patients with right-side breast cancers, 4D-CT scans were performed using the free-breathing scan protocol. In this scenario, the maximum respiratory motion was restrained not to exceed 1.5cm; the respiratory rate was maintained at about 13 times per minute, and the optimal scanning pitch was set based on our previous studies (27). Furthermore, the contouring of ROIs and the extraction of the radiomics features were conducted in the MIP image mode. Therefore, the impact of respiratory motion on the training and verification of the machine learning model should be negligible.

Although the prediction model of this study requires further validation on an additional center as an independent test, we believed that the partition of the dataset into training set and validation set is good practice to ensure the reliability of the predictive models developed. In building GBDT model, we used the internal data cross-validation method (i.e., 75% of patients as the training set, and the remaining 25% as the validation set). Given the small sample size, this cross-validation method can make full use of the data. This internal cross-validation method may be more suitable for small sample dataset and can improve the generalization ability of the model, as reported in previous studies on machine learning applications (28, 29). Part of procedures of this method is similar to that reported previously by Kocak et al. They performed feature extraction and dimensionality reduction on CT images of all patients before adopting a 10-fold cross validation random forest training and validation (30). In our future work, we will consider to combine the dataset of our center with other regions in China, in which an independent test cohort can be obtained to achieve improved reliability of the prediction model.

Inflammatory response has been shown to be generally associated with RD 2+. In the initial period of RT, there is an immediate generation of an inflammatory response. The early inflammatory response to radiation is mainly caused by pro-inflammatory cytokines (e.g., IL-1, IL-3, IL-5, IL-6, and tumor necrosis factor [TNF]-a), chemokines, receptor tyrosine kinase, and adhesions molecules. These factors can create local inflammatory response of eosinophils and neutrophils. Janko et al. have ascertained that IL-1 had an important role in the development of RD 2+. They found that mice that lack either IL-1 or the IL-1 receptor developed less inflammation and less severe pathological changes in their skin (31). On the other hand, 80% of tissues and cells are composed of water. Most of the radiation damage from exposure of low-LET rays is due to the radiolysis of water resulting in the production of free radicals (ROS) and reactive nitrogen species (RNS). Radiation leads to an upregulation of free radicals and oxidases in tissues, and the distributions of which in cells, tissues and organs are heterogeneous.

Given these facts, we expect that the distributions of pro-inflammatory cytokines, ROS and RNS in the skin are individualized and specific in patients, and these specificities or differences might be reflected by the different distributions of radiomics features, such as distributions of the feature values of IH_Gauss Fit1 Gauss Mean, GLCM_25225.4Contrast, and IH_Gauss Fit1 Gauss_Std shown in **Figure 5**. The specific relationship between the distributions of cytokines and enzymes and radiomics signatures needs to be further investigated.

As can be observed in **Figure 5**, the high values of IH_Gauss Fit1 Gauss Mean feature in PTV_100PD of the patient with RD 2+ mainly appeared close to the body surface and chest wall, and distributed in strip pattern. Whereas the high value of this feature in the patient without RD 2+ appeared in the middle of PTV_100PD in a cluster style. The GLCM_25225.4Contrast feature has a scatter-like distribution in the SKIN_30Gy of the patient with RD 2+, whereas the feature of the patient without RD 2+ has a single-hot-spot distribution. The IHGaussFit1GaussStd feature has little difference in the heat map within SKIN_30Gy; however, the histograms (i.e., amplitude distribution) of the feature values between the patient with and without RD 2+ exhibit apparently different envelopes. These exemplary distributions of radiomics features between patients with and without RD 2+ demonstrated their potential to identify the patients at the high risk of RD 2+. However, the correlation between the occurrence location of RD 2+ and the spatial distribution of radiomics feature needs to be further investigated in the future study. We envision that the prediction of the locations where RD 2+ occurs in advance of RT will be possible, which would facilitate personalized skin care prior to the occurrence of severe RD 2+.

## 5 Conclusion

In this study, we developed a novel dose-gradient based GBDT machine learning model using 20 CT radiomics features within PTV_100PD, PTV_105PD, PTV_108PD, SKIN_20Gy and SKIN_30Gy volumes and 8 clinical and dosimetric characteristics to predict RD 2+ in breast cancer patients before radiotherapy treatment. Our results demonstrated that combining features within multiple ROIs related to different dosimetric gradient in treatment planning CT images can achieve the best prediction performance compared to using single ROI as well as clinical or dosimetric characteristics only. The model offers the opportunity to take the risk of RD 2+ and the sensitivity of multiple ROIs into account in the radiation therapy planning procedures, thus enabling the personalized radiation dose distribution at the planning stage of RT to improve outcomes for patients at high risk for RD 2+.

## Data availability statement

The original contributions presented in the study are included in the article/supplementary material. Further inquiries can be directed to the corresponding authors.

## Ethics statement

The studies involving human participants were reviewed and approved by Medical Ethics Committee of Hangzhou Cancer Hospital. The patients/participants provided their written informed consent to participate in this study.

## Author contributions

XL and YK created the study design. HF, QN, ZY, LX and YR collected the clinical and CT data and processed the data. HF, XL and HW conducted data analysis. XL and HW wrote the manuscript. SM, QD, XC and BX gave suggestions regarding the radiodermatitis grading. All authors contributed to the article and approved the submitted version.

## Funding

This study was supported by the Natural Science Foundation of Zhejiang Province, China (LGF22H220007), Hunan Provincial Natural Science Foundation, China (2022JJ30976), Hangzhou Health Science and Technology Project, China (A20200746).

## Conflict of interest

The authors declare that the research was conducted in the absence of any commercial or financial relationships that could be construed as a potential conflict of interest.

## Publisher’s note

All claims expressed in this article are solely those of the authors and do not necessarily represent those of their affiliated organizations, or those of the publisher, the editors and the reviewers. Any product that may be evaluated in this article, or claim that may be made by its manufacturer, is not guaranteed or endorsed by the publisher.
